# Education on organ donation and transplantation in primary school; teachers' support and the first results of a teaching module

**DOI:** 10.1371/journal.pone.0178128

**Published:** 2017-05-22

**Authors:** Marion J. Siebelink, A. A. Eduard Verhagen, Petrie F. Roodbol, Marcel J. I. J. Albers, Harry B. M. Van de Wiel

**Affiliations:** 1 Groningen Transplant Center, University Medical Center Groningen, University of Groningen, Groningen, The Netherlands; 2 Department of Paediatrics, Beatrix Children’s Hospital, University Medical Center Groningen, University of Groningen, Groningen, The Netherlands; 3 Hanze University of Applied Sciences Groningen, University Medical Center Groningen, Groningen, The Netherlands; 4 St. Elisabeth Hospital, Tilburg, The Netherlands; 5 Wenckebach Institute for Medical Education, University Medical Center Groningen, University of Groningen, Groningen, The Netherlands; TNO, NETHERLANDS

## Abstract

Organ and tissue donation can also involve children. Because of its sensitivity, this topic requires careful decision making. Children have the ability to carefully reflect on this subject and enjoy participating in family discussions about it. Therefore, what children need is proper information. When schools are used to educate children about this subject, information about teacher support for this type of lesson along with its effects on the depth of family discussions is important. Methods: A questionnaire was sent to all 7,542 primary schools in the Netherlands. The goal was to gather information on teachers’ perspectives about a neutral lesson devoted to organ and tissue donation, and also on the best age to start giving such a lesson. The second part of our study examined the effects of a newly developed lesson among 269 primary school pupils. The school response was 23%. Of these, 70% were positive towards a lesson; best age to start was 10–11 years. Pupils reported 20% more family discussions after school education and enjoyed learning more about this topic. There is significant support in primary schools for a school lesson on organ and tissue donation. Educational programs in schools support family discussions.

## Introduction

Donation and transplantation are discussed predominately in the context of care for adults [1]. When family members must decide about organ donation, as surrogate decision-makers, knowledge of the deceased’s intentions influences their decision [2–4]. Most parents do not discuss organ donation with their children although children are willing to discuss this topic [5]. As a consequence, parents often have to make a donation decision for their child, without any direct knowledge about their child’s intention or their opinion about organ donation [4,6,7]. This is more appropriate as in some countries (e.g. The Netherlands) children of 12 years and older are considered legally capable in this topic.

The best way to provide a family with insight in how the child thinks about organ donation, is by having a discussion about this with the family in a non-crisis situation [8]. We presume that these discussions are likely to occur more often if awareness of organ donation among children increases.

Good information is a prerequisite for a valuable and balanced family discussion about organ donation. Several authors and organizations have argued that it is the responsibility of the government to provide the public with clear information about organ and tissue donation, and to educate children about donation [6,9,10,11]. Although these school educational programs about organ donation seem to focus mainly on high school students, studies report that children as young as 9 years and older have heard about organ donation through social media and other internet sources [5]. This implies that in order to have good family discussions, we need to provide children with age-specific neutral information. Educational programs on organ donation in primary schools could be useful tools to achieve that goal. Moreover, these educational programs might also increase knowledge and awareness among parents and other family members. This raises the question of the extent to which school teachers also feel a certain need for an educational program about organ donation. Do teachers support education about this subject and what would be the effects of this lesson on family discussions?

To answer these questions, we conducted a study aimed, first, at evaluating the opinion of primary school teachers about lessons in their school classes about organ and tissue donation and transplantation, and about the preferred age and method to educate children about this subject. The second aim of this study was to find out what the learning effects of these lessons on the children were and to what extent the lessons contributed to the family discussions on organ and tissue donation.

## Materials and methods

The study design was twofold. Part A contained a questionnaire which was sent out to all principals of primary schools (pupils 4–12 y/o) in the Netherlands. The questionnaire had been tested through interviews at five primary schools in the Netherlands. On the basis of these interviews, the final questionnaire was divided into two perspectives: an “informative” (What are organs and tissues, and what is transplantation?) and a “forming-an-opinion” (What is donation? What do I think about it myself?) perspective.

In April 2008, the questionnaire was sent to all 7,452 primary school principals-teachers in the Netherlands, along with a self-addressed envelope. In the Netherlands it is common that the principal is also a part-time teacher. The time allowed for response was four weeks. The questionnaire contained the demographic variables of the respondent (sex, age, function, teaching experience) and the religious affiliation of the school. In addition the questionnaire consisted of questions related to donation and transplantation (see Table 1). Taking into account that in large national surveys it is expected that 8% of the questionnaires sent out prove undeliverable for various reasons (e.g., merger or closing of the school), we assumed that 6,856 (of the 7,452) questionnaires would actually reach the primary schools.

**Table 1:** Part A: Questions related to donation and transplantation for primary school principals-teachers.

Question	Possible answers
Are you familiar with the subject of organ donation?	yes/no
Are you registered in the Dutch Donor register?	yes/no/unknown
How are you registered? (optional)	consented/refused/decision up to relative
Do you have teaching experience with the subject?	yes/no
Do you have sufficient knowledge about the subject?	yes/no
Do you want to give a lesson on this subject?	yes/no
If yes, what age groups should receive an informative lesson?	age groups
If yes, what age groups should be forming an opinion?	age groups
If not, what is the reason?	free field
Do you use any educational program on this subject at school?	yes/no
In what kind of lesson?	biology/social sciences/others
What kind of teaching methods do you prefer?	free field
Is it desirable to explicitly inform parents about this lesson?	yes/no

Following the results of the questionnaire for the school principals/teachers (part A) of this study, an educational lesson on organ and tissue donation (www.donordenkers.nl) was developed. This lesson was supported by the Dutch government, in cooperation with the National Transplant Foundation and a specialized bureau for digital education including active support from a panel of teachers. The release of this lesson was in 2010.

Part B contains a pilot study of the pupils to the effects of this lesson by conducting a quantitative questionnaire survey. In 2011 we invited, by telephone 8 primary schools in the Netherlands to participate, in a stratified random sample taking their religious affiliation into account as demographic variable. The questionnaire was aimed at children in the two highest grades of primary school (10–12 years old) and was administered by a researcher in the classroom. The questionnaire was administered at three different moments in time. The first, T0, was administered before the educational lesson about organ and tissue donation and transplantation was given. T1 was administered directly after the approximately 1.5-hour lesson and was overseen by the teacher; T2 was administered three weeks later. The questionnaires contained questions related to knowledge (see Table 2) and questions about family communication about the topic. Next, we asked the pupils for their opinions about this type of lesson.

**Table 2:** Part B: Questions related to knowledge for primary school pupils.

Questions	Possible answers	
1-What is donation?	□ donation is receiving □ donation is giving	
2-If you are a donor then…	□ you can choose to whom you give your organs □ you can only give organs to your own family □ you cannot choose the recipient(s)	
3-You can donate if you are deceased and if…	□ you are younger than 30 years old □ you are older than 30 but younger than 60 years □ you are older than 60 □ age doesn’t matter	
4-Check off the organs that can be donated	□ kidneys □ liver □ heart	□ lungs □ bones □ aorta
5-Check off the tissues that can be donated	□ bone tissue □ heart valve □ pancreas	□ skin □ intestine (bowel) □ cornea

The data were analysed with SPSS 17 using the chi-squared tests (SPSS Inc., Chicago IL, USA). A p-value less than .05 was considered statistically significant. According to Dutch law a survey study like this does not require approval by a medical ethical review board, which was confirmed by the medical ethical review board of the University Medical Center Groningen. No consent of the participants was needed since the data were analysed anonymously.

## Results

### Part A; the school principals-teachers

Of the questionnaires, 1,582 (23%) were returned within four weeks. The main religious affiliation of the respondents corresponded with the distribution of primary schools in the Netherlands. Of the respondents, 61% were men, 39% women; 17% were younger than 40 years old, 14% were between 41 and 50 y/o, and 61% were older than 50 y/o. Most of the respondents (1,270/80%) were school principals who are also part-time teacher, 14% were teachers, and 6% were individual supervisors. Eighty-eight percent had more than 10 years of teaching experience.

Of the respondents, 65% were personally registered in the Dutch Donor Register, and 80% of these answered as to how they were registered: 59% were willing to become donors, 6% refused donation, and 15% thought that it was up to relatives to decide.

There was little teaching experience in schools concerning the subject of organ donation; in 80% of the schools, organ donation had never been discussed in the classroom before. The 20% who did have teaching experience answered that attention had been paid to the subject, because it was part of their teaching method (8%) or because one of the school pupils or one of their relatives had needed a transplant or had become a donor (12%). All of these teachers stated that they were strongly in need of teaching materials to support this lesson.

To the question of “Do you want to give a lesson on this subject?” 70% answered positively. Twenty-eight percent of the respondents did not want to give a lesson on organ donation, and 2% answered that they did not know. Respondents who were negative towards a lesson mostly mentioned that they thought that their children were “too young to discuss this subject,” “that this was a subject to discuss only at home,” or that they thought that the school curriculum was full enough.

A significant relationship was found between a respondent’s own registration and a positive attitude towards a lesson (X ^2^, p = .015). Religion had no significant relationship with opinions about a lesson on organ donation (X ^2^, p = .328), although a certain tendency did seem to exist; schools with a religious affiliation were more often negative towards a lesson about organ and tissue donation. Age, sex, and function, as well as teaching experience were also not related to attitudes concerning a lesson on this subject.

**Fig 1:**
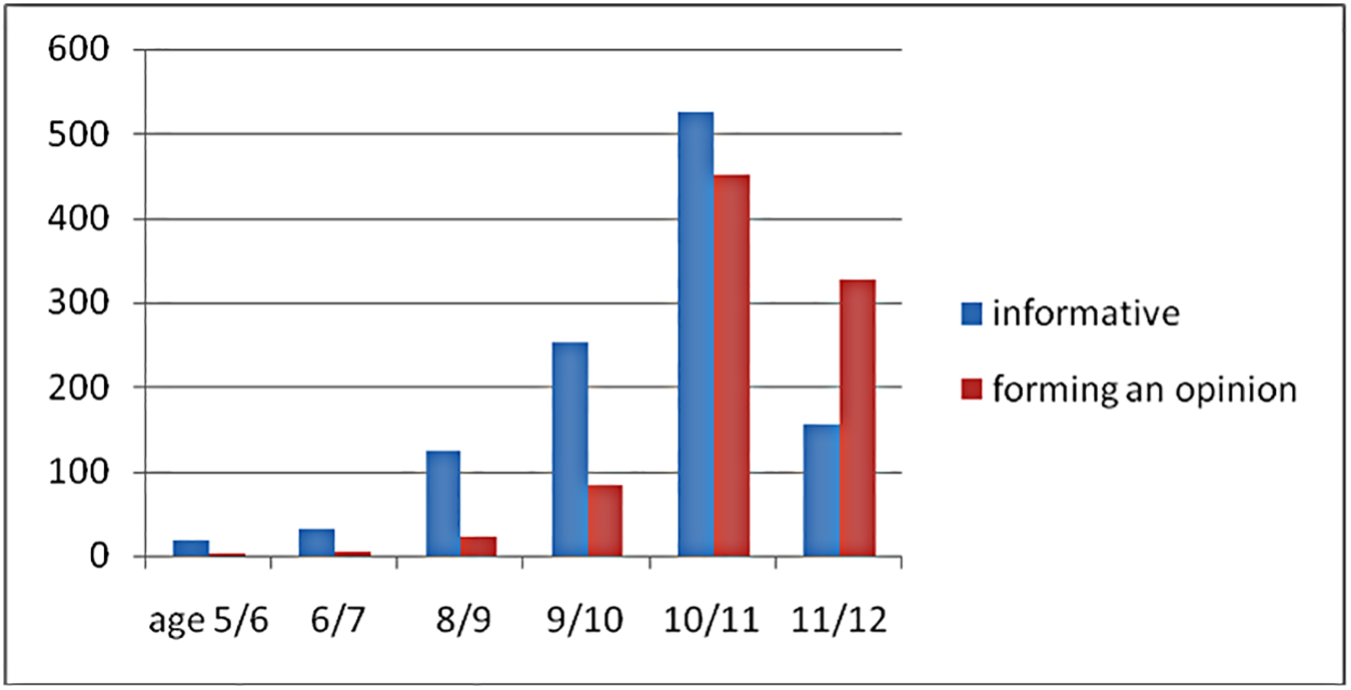
Best age to start a lesson on organ donation. Vertical axis: Number of positive responding teachers. Horizontal axis: the best age to start an informative lesson and a lesson in forming an opinion.

Fig 1 shows that teachers thought the best age for a first informative lesson was around 9-11 years old; most teachers (57%) thought a biology lesson was the best occasion. The best age for a first lesson aimed at forming an opinion was around the ages of 10–12 years. According to the respondents, this could best be incorporated in a social studies lesson (22%) or in a group discussion. In addition, a combination of both themes was thought possible in a lesson on public health issues. Suggestions as to how to develop a lesson on the topic of organ and tissue donation were given in 964 cases (e.g., first-aid lessons, lecture, writing an essay).

Most of the respondents (76%) preferred for the parents to be explicitly informed about this lesson. However, 13% thought that it was not necessary to inform the parents at all; they considered that this was a part of normal socially oriented education.

Respondents who were positive towards a lesson on organ and tissue donation in school more often felt that they needed more information to be able to give this lesson than did those respondents who were negative towards a lesson on this subject. These latter teachers were more often likely to answer that they already knew enough about this subject.

### Part B; the students

The school-based digital lesson educated children about transplantation and informed children in a neutral way about donation. It also contained an extensive teachers’ manual how to give this lesson.

In measurements T0 and T1 of the study, all 269 invited children participated (mean age 10.9 years); 125 children were in grade 7 (10–11 y/o) and 144 children in grade 8 (11–12 y/o).

**Fig 2:**
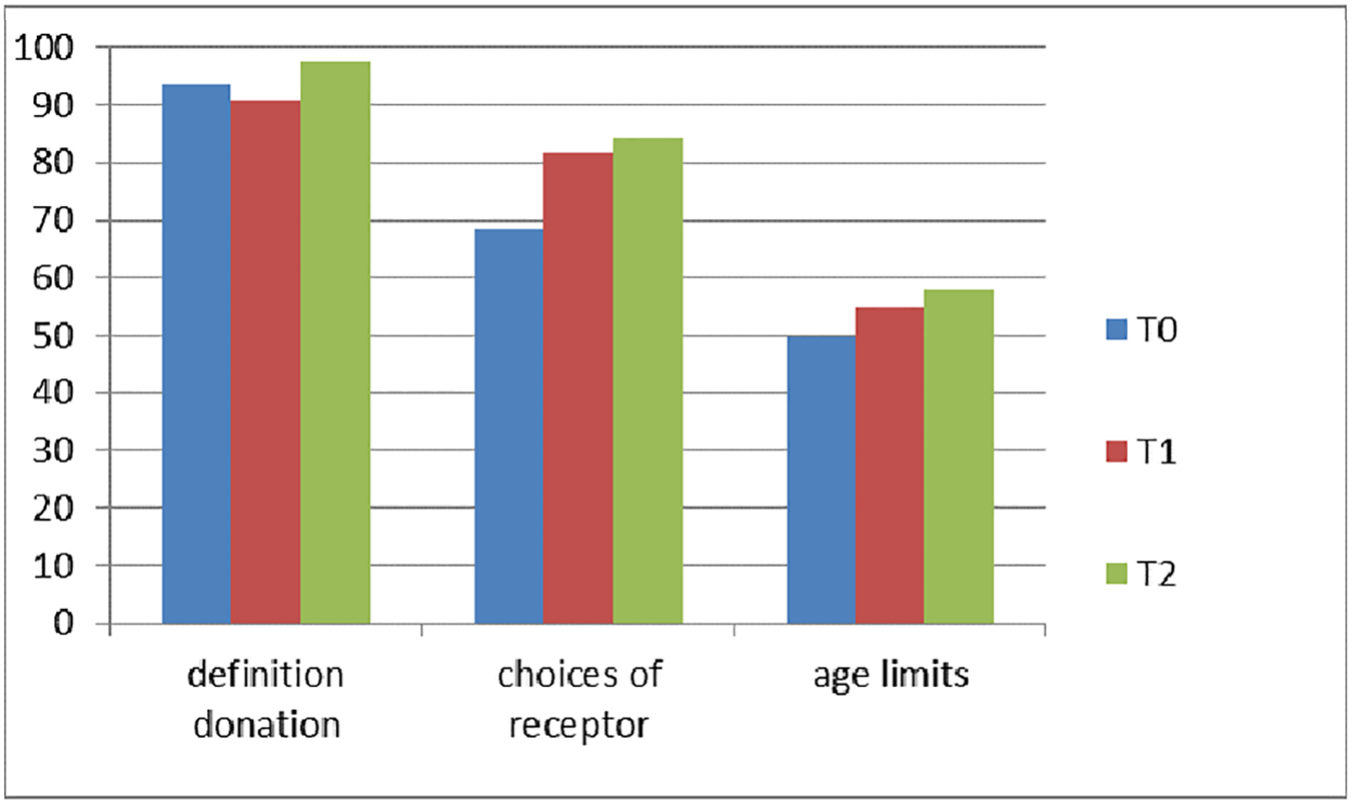
Children’s knowledge about organ donation. Percentage of correctly answered questions.

In T2, the last questionnaire, 257 children participated (12 children were on excursion). The results of questions 1, 2, and 3 are shown in Fig 2.

Table 3 shows the answers on questions 4 and 5: specific knowledge about organs and tissues at the three measuring moments. The knowledge, especially directly after the lesson, improved significantly. Although this knowledge decreases at T2, there is an overall increase of knowledge between T0 and T2.

**Table 3:** Specific knowledge of the students about organs and tissues in the measurements.

	% (sd) correctly answered in questionnaire			Differences before and after lesson	Differences directly after lesson and 3 weeks later
	T0, T1, T2				
	T0	T1	T2	Significant change	Significant change
				T0-T1	T1-T2
**kidney**	93 (0.26)	99 (0.06)	96 (0.20)	p < .001	p = .01
**lung**	85 (0.35)	92 (0.27)	84 (0.36)	p = .02	p = .01
**liver**	76 (0.42)	91 (0.29)	87 (0.32)	p < .001	P = .23
**heart**	76 (0.41)	96 (0.18)	85 (0.36)	p < .001	p < .001
**bone**	28 (0.45)	57 (0.49)	54 (0.50)	p < .001	p = .47
**heart valve**	60 (0.49)	76 (0.43)	79 (0.41)	p < .001	p = .35
**skin**	52 (0.50)	85 (0.35)	81 (0.37)	p < .001	p = .30
**cornea**	39 (0.49)	78 (0.41)	65 (0.48)	p < .001	P < .001

**Fig 3:**
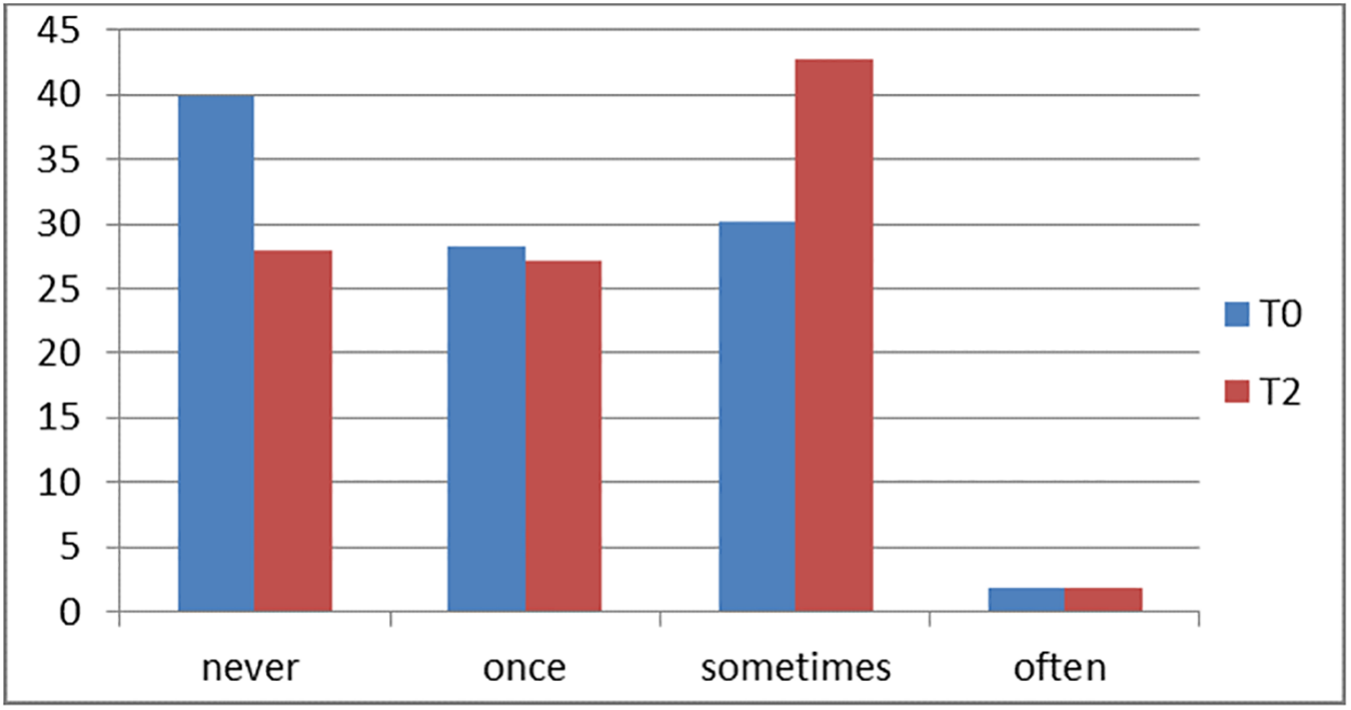
Family discussions about donation. Percentage before and three weeks after a lesson about organ donation.

Fig 3 shows the number of times children discussed the topic at home, before the lesson was given (T0), and three weeks after the lesson (T2). Before the lesson was given, 60% of the children had discussed the topic at home once or more often. Of the 40% of children who had never discussed this topic at home, 8% answered that they were not familiar with the topic. Most of these children answered that they had never thought about it on their own. Three weeks after the lesson, 72% of the respondents had discussed the topic at home, which was significantly higher (X ^2^, p = .04). For children who had never discussed the topic at home, this lesson was the reason that this family discussion started.

After the lesson, children were asked to give their evaluation, on a five point scale, of such a lesson. Twenty-six percent found the lesson very good, 41% found it good, 26% of the respondents were neutral, 5% did not like the lesson, and 2% found it terrible. Of all respondents, 70% stated they would like to learn more about this topic later on in high school.

## Discussion

We wanted to know the extent to which primary school teachers feel the need for an educational program about organ donation and we examined the effects that such a lesson would have on children in terms of increase of knowledge and family discussions with the children. As far as we know, this is the first study focusing on this topic.

Of the teachers 70% were willing to discuss this subject in their classrooms. We investigated factors that could potentially be associated to support for a lesson on organ donation in school. The registration rate of those respondents who were in the Dutch Donor Register (65%) was higher than the average percentage of registrations in the Dutch Donor Register for the population at large (44%). It is conceivable that teachers are more involved in social and public health issues and therefore are more willing to register their wishes. Meanwhile, the rate of positive registration of all registered respondents (59%) is comparable with the 57% found in the Dutch Donor Register [12].We found a significant relationship between a positive registration by the respondent and a positive attitude towards a lesson on this subject at school. This was not a surprising finding for us, because we presume that teachers’ personal ideas about public health issues may have an influence on what subjects are discussed in the classroom.

We found that most teachers did not have any actual teaching experience with the subject. However, it is interesting to note that 75% think that they already have enough knowledge about the subject. We think that this is a comforting finding because we are convinced that a teacher’s specific knowledge is a prerequisite for good lessons on organ and tissue donation in school.

It is desirable, according to most of the respondents, to inform parents that this lesson is being given. This finding seems to imply that when parents are informed about a lesson taking place, they should also simultaneously receive information about the subject; this could serve as an aid for family discussions at home. Therefore, we think that school can serve as a basis for initiating these family discussions, and we already know how important it is for these discussions to take place in a non-crisis situation [7]. Overall, it was hypothesized that this educational program would aid family discussions.

Part B of our study tested this hypothesis and tested children’s knowledge before and after a lesson about donation and transplantation. In this pilot study we found a surprising amount of knowledge in T0 for which we have no clear explanation. Probably this was because in one school (two classes) there had been a child with a liver transplantation. Generally speaking, children’s knowledge about the topic appeared to increase after the lesson was given and most of this knowledge still existed after 3 weeks. As we were not able to compare our findings with other studies about education on donation, more research is needed to put these findings into perspective. Next to this it is important to measure the long term knowledge and how the knowledge contributes to donation attitudes. Overall, our findings seem to underline that proper information is important for children in order to further realistic ideas about this sensitive topic. We think that it is of great importance to repeat an age-appropriate lesson on organ donation in the secondary school. This will contribute to long term knowledge and attitude differences and we expect that this will also contribute to better registration rates in the Dutch donor register.

The most important result was that we found that children significantly discussed the topic at home more often after the lesson. We therefore think that the child’s education on organ donation and transplantation is an important factor in stimulating family discussions. These family discussions are likely to increase parental insight into the child’s reasoning and provide access to the child’s views about organ and tissue donation. Since these findings are a first indication, they may need confirmation in future studies. Some limitations are worth noting. Although the response rate in part A is sufficient, we do not know the reasons for the non-response. It is possible that the results are biased due to respondents who are positive towards a lesson being more willing to fill in the questionnaire. On the other hand, the registration type of the respondents in the Dutch Donor Register is comparable with the registration of the general public. The results in part B could have been influenced to an extent because one school had had experience with a transplantation. Although this study focused on the situation in the Netherlands, we have no reason to suppose that it could not be extrapolated to other countries.

### Conclusion; translation to health education practice

We found sufficient support for a lesson on donation and transplantation, and the majority of the children appreciated this type of lesson. These findings support the idea of developing a curriculum on organ donation that begins in the highest grades of primary school and continues into middle/junior and high school. This curriculum would contribute to health literacy on this topic, and provide children and their families with appropriate information in order to make proper decisions about donation.

## Supporting information

S1 File Teacher’s data. (SAV)

S2 File Children’s data. (SAV)
